# Photo-responsive functional materials based on light-driven molecular motors

**DOI:** 10.1038/s41377-024-01391-8

**Published:** 2024-03-01

**Authors:** Yanping Deng, Guiying Long, Yang Zhang, Wei Zhao, Guofu Zhou, Ben L. Feringa, Jiawen Chen

**Affiliations:** 1 SCNU-UG International Joint Laboratory of Molecular Science and Displays, National Center for International Research on Green Optoelectronics, South China Normal University, Guangzhou, 510006 China; 2 Stratingh Institute for Chemistry, University of Groningen, Nijenborgh 4, 9747AG Groningen, The Netherlands

**Keywords:** Optics and photonics, Other photonics

## Abstract

In the past two decades, the research and development of light-triggered molecular machines have mainly focused on developing molecular devices at the nanoscale. A key scientific issue in the field is how to amplify the controlled motion of molecules at the nanoscale along multiple length scales, such as the mesoscopic or the macroscopic scale, or in a more practical perspective, how to convert molecular motion into changes of properties of a macroscopic material. Light-driven molecular motors are able to perform repetitive unidirectional rotation upon irradiation, which offers unique opportunities for responsive macroscopic systems. With several reviews that focus on the design, synthesis and operation of the motors at the nanoscale, photo-responsive macroscopic materials based on light-driven molecular motors have not been comprehensively summarized. In the present review, we first discuss the strategy of confining absolute molecular rotation into relative rotation by grafting motors on surfaces. Secondly, examples of self-assemble motors in supramolecular polymers with high internal order are illustrated. Moreover, we will focus on building of motors in a covalently linked system such as polymeric gels and polymeric liquid crystals to generate complex responsive functions. Finally, a perspective toward future developments and opportunities is given. This review helps us getting a more and more clear picture and understanding on how complex movement can be programmed in light-responsive systems and how man-made adaptive materials can be invented, which can serve as an important guideline for further design of complex and advanced responsive materials.

## Introduction

Nature has provided a large collection of molecular machines and devices that are among the most amazing nanostructures on this planet. As seen for instance in the process of vision, the ATP synthase rotary motor function, or the photosynthesis in the green plant these dynamic molecular systems are able to sustain responsive, adaptive and complex biological processes which are key to proper functioning of our organisms and enable out-of-equilibrium operation of biological systems^1–8^. These processes are accomplished with high efficiency and selectivity under precise control at the molecular level. Inspired by these sophisticated natural molecular machines, scientists have been working on designing and constructing artificial molecular machines with different dynamic functions via synthetic approaches. In the past two decades, the research and exploration of molecular machines has mainly focused on developing molecular systems at the nanoscale^9–16^. Through elegant molecular design and effective organic synthesis, molecular switches^17–20^, molecular motors^21–29^, molecular rotors^30–33^, molecular pumps^34–39^, molecular cars^40–44^, and molecular assembly lines^45,46^ have successfully been demonstrated. At the nanoscale, the designed functions in these molecular machines make them responsive to external signals, such as light^47–52^, electricity^44,53,54^, heat^55–57^, magnetism^58,59^, pH^60–64^, etc., and provide precisely defined controllable mechanical output. With the rich development in design and synthesis at the molecular level, a key scientific issue should be addressed i.e. how to amplify the controlled motion of molecules at the nanoscale along multiple length scales, such as the mesoscopic, or the macroscopic scale, or towards a more practical perspective, how to convert molecular motion into changes of properties of a macroscopic material. A most straightforward approach is to build molecular machines inside the material through supramolecular self-assembly or covalent bonding^65–69^. Taking inspiration by this idea of embedding intrinsic motor functions into materials, scientists have successively developed various multicomponent responsive materials, accelerating major developments in the field of organic functional, mechanical and smart materials.

In recent years, the use of light as the external stimulus and as a clean energy source has received much attention^70,71^. As light can be precisely controlled with short response time, produces no waste and high spatial and temporal precision can be reached, photo-responsive smart materials have seen major developments. Among the examples that have been reported so far, most cases are based on two typical molecular photoswitches: azobenzene and diarylethene. After being irradiated at the appropriate wavelength of light, azobenzene can undergo *trans*–*cis* isomerization^72,73^, while diarylethylene can undergo ring opening or closure reactions^74–76^. The molecular configurations of these two types of molecular photoswitches are changed upon irradiation, resulting in major changes in the shape, polarity and electrical properties of the entire molecule. These changes are the key to dynamically tuning the properties of smart materials^77–82^.

**Fig. 1:**
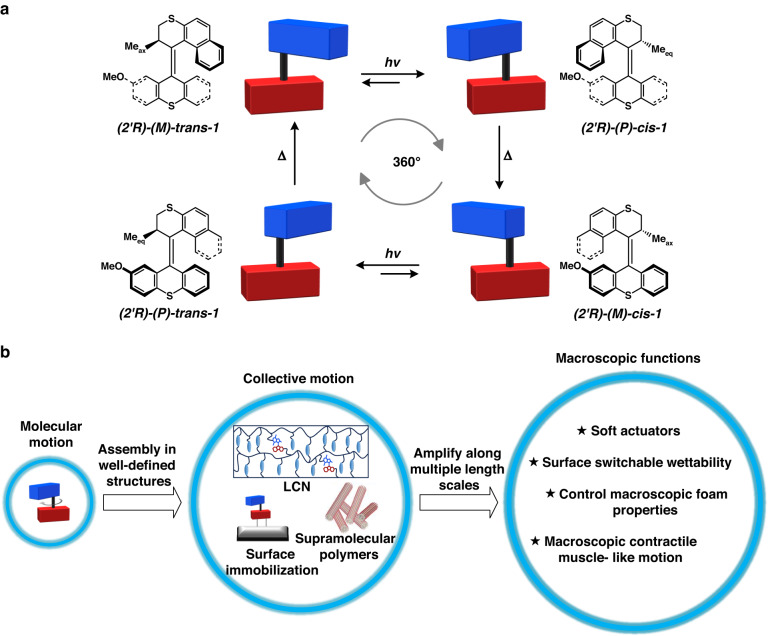
Photo-responsive functional materials based on light-driven molecular motors. **a** Chemical structure of light-driven molecular motor **1** and schematic representation of its 4-step rotary process. Adapted with permission^83^. Copyright © 2000 American Chemical Society. **b** Schematic illustration of the amplification of rotary motion of molecular motors along length scales to achieve responsive macroscopic functions

However, these two classes of molecular switches only have two or three static states, making it impossible for the entire system to reach out of equilibrium that can lead to repetitive or continuous motion, and therefore are limited in mimicking the sophisticated dynamic properties of the materials found in biological systems. Among all the photo-responsive molecular machines, light-driven molecular motor based on overcrowded alkenes are excellent candidates as these can induce continuous motion that can lead photo-responsive materials into a new era. Figure 1a shows a representative structure of a unidirectional rotary molecular motor^83–88^. The upper half of the motor can be considered as a rotator and the lower half as a stator, while the central double serves as the rotary axle. Stable *(M)-trans-***1** adopts a specific intrinsic helicity due to the significant steric crowding present in the structure.

The methyl group at the stereogenic center adopts a pseudo-axial orientation to minimize steric interaction with the lower half of the motor. A photochemical *trans*–*cis* isomerization of the central double bond takes place when stable *(M)-trans*-**1** is irradiated by UV light (λ = 365 nm). During this isomerization, the rotor part rotates anti-clockwise with respect to the stator part, resulting in unstable *(P)-cis*-**1** in which the overall helicity of the molecules is changed. The *(P)-cis*-**1** is a thermally unstable isomer since the stereogenic methyl group is forced to adopt an unfavorable pseudo-equatorial orientation, which pushes it towards the lower half. To release the steric strain, an irreversible thermally activated step occurs where the methyl group and the naphthalene upper half slip past the aromatic parts of the lower half, generating stable *(M)-cis*-**1**. This step is accomplished by an inversion of the helicity of the molecule and allows the stereogenic methyl group to regain the favored pseudo-axial orientation. Another photo-induced *cis–trans* isomerization which is followed by a thermal helix inversion step completes the unidirectional 360° cycle. The directionality of the rotation is controlled by the absolute configuration of the stereogenic center and the use of the other enantiomer leads to the opposite rotary direction. Alternation of structure of the motor offers several practical advantages, including selection of motors with a large collection of different rotary speeds and tuning of the wavelength of irradiation^30,85,89–95^.

Taking light-driven molecular motors as a tool to control molecular motion and to build up advanced functional multicomponent molecular systems has been proved to be successful and several reviews have discussed the design, synthesis and operation of the elegant mechanical systems at nanoscale^20,84,85,90,96–99^. However, photo-responsive macroscopic materials based on light-driven molecular motors, which serve to demonstrate important applications of such motors, have not been comprehensively summarized. Therefore, in the present mini-review, the key approach to amplify the molecular motion is to take advantage of the collective motion and to achieve macroscopic functions including dynamic control of surface wettability, muscle-like functions, directional movement and helical coiling (Fig. 1b). We first discuss the strategy of confining relative molecular rotation into absolute rotation by grafting motors on surfaces. Secondly, the cases of self-assembled motors in supramolecular polymers with high internal order will be illustrated. Moreover, we will focus on building of motors in a covalently linked system such as polymeric gels and polymeric liquid crystals to generate complex dynamic functions. Finally, a perspective toward future developments and opportunities is presented.

## Dynamic control of surface wettability

Motors operated in solution are facing random Brownian motion, which makes it difficult to gain both positional and orientational order of the molecules. Therefore, cooperativity of rotary motors is largely prevented and it is hard to harness useful work. One possible solution to overcome this problem is to immobilize the motors on a surface, converting the relative rotation of one part of the molecule with respect to the other part to absolute rotation relative to the surface. This surface confinement allows to achieve efficient organization and orientation, and taking advantage of the light-responsive rotational motion of the molecular motors enables them to regulate surface properties^22^.

**Fig. 2:**
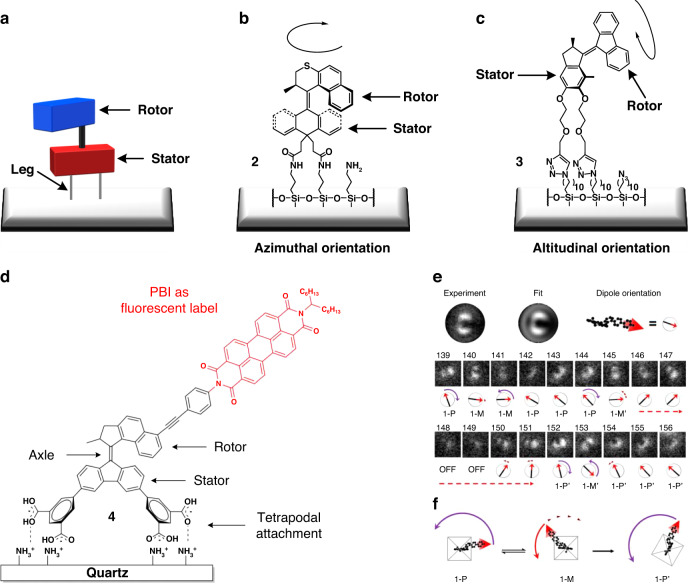
**Approaches to anchor molecular motors to surfaces. a** Schematic illustration of molecular motor immobilized on surface. **b** Motor **2** in an azimuthal orientation. Adapted with permission^100^. Copyright © 2007 WILEY‐VCH Verlag GmbH & Co. KGaA, Weinheim. **c** Motor **3** in an altitudinal orientation. Adapted with permission^101^. Copyright © 2009 The Royal Society of Chemistry. **d** First design of a PBI-Labeled Surface-Bound Molecular Motor **4**. Reproduced with permission^103^. Creative Commons CC BY-NC-ND 4.0 License, 2018, published by American Chemical Society. **e** Experimental defocused patterns of surface-bound motors were fitted to a library of calculated patterns to derive the in-plane and out-of-plane angles describing the orientation of the dipoles and thus the relative nanomechancial state in each frame. **f** Proposed interpretation of the optomechanical response for surface-bound motors. Reproduced with permission^104^. Copyright © 2017 American Chemical Society

A key step is the assembly of the rotating motors on surfaces and their integration with macroscopic systems. Molecular motors with distinct upper and lower halves incorporate the capability to be attached on surfaces introduce various ‘legs’ for surface anchoring within the stator component, thereby allowing the rotor component to execute light-driven rotational motion freely (Fig. 2a). In addition, more than two attaching points are needed for stable orientation of the motors on surfaces and to provide enough free volume for the individual photoactive part to perform the desired rotational motion. Figure 2b, c show the characteristic approaches of assembly of motors on surfaces and they can be categorized into azimuthal^100^ and altitudinal^101,102^ rotations based on the direction of rotational motion with respect to the surface upon attachment. Motors that rotate in an altitudinal direction with respect to the surface are expected to have higher potential for dynamic control of the properties of surfaces because the exposure of functional groups on the rotor can be modulated in a cyclic manner. In addition, we have incorporated a fluorescent tag to the azimuthal anchored motor^103^ (Fig. 2d). In a joined effort with the Hofkens group, we are able to monitor the unidirectional movement of a motor at single molecular level by employing wide field defocused fluorescence microscopy (Fig. 2e, f)^104^.

**Fig. 3:**
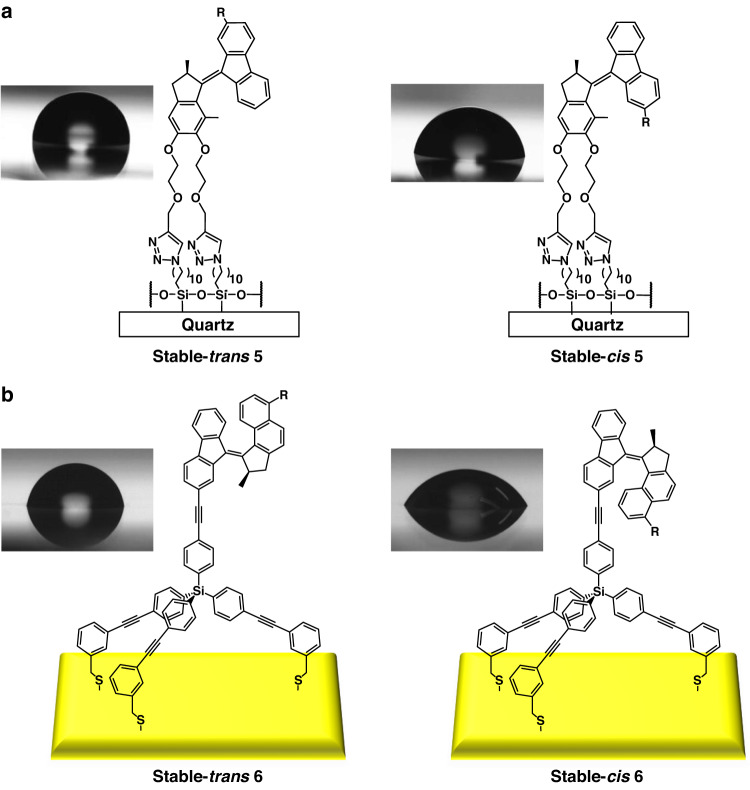
**Control of surface wettability by molecular motors. a** Schematic representation of bipodal motor **5** immobilized on a quartz surface and the switchable wettability of the self-assembled monolayers (SAMs). Reproduced with permission^105^. Copyright © 2013 WILEY‐VCH Verlag GmbH & Co. KGaA, Weinheim. **b** Schematic representation of tripodal motor **6** immobilized on a gold surface and the switchable wettability of the SAMs. (**R** = **C**_**4**_**F**_**9**_). Adapted with permission^106^. Copyright © 2014 American Chemical Society

Our group has designed altitudinal motors with rotor parts functionalized with hydrophobic perfluorobutyl units attached to quartz or gold surfaces via their stator parts^105,106^, as shown in the Fig. 3. Through UV-Vis experiments, it was confirmed that the motors could operate properly both in solution and on surfaces, and contact angle measurements on *cis* or *trans* modified surfaces confirmed that they have different wettability properties depending on the polarity and orientation of the substituent groups, triggered by the rotary cycle of the motor. It is worth noting that the fluorinated chain reduces the free volume in the interface, thus minimizing the interaction between the water and hydrophilic components, and when switching on and off the surface wettability change is enhanced. In addition, the tripodal attachment shows more profound effect than that of the bipodal attachment. This is attributed to the fact that the tripod structure creates enough free volume between the rotors to enhance the high degree of perpendicular orientation (with respect to the surface), which prevents motor interactions within the self-assembled monolayers as well as direct interactions with the underlying gold substrate, resulting in effective photo-induced reorganization of surface structure (Fig. 3b).

## Photoresponsive supramolecular polymers based on molecular motors

**Fig. 4:**
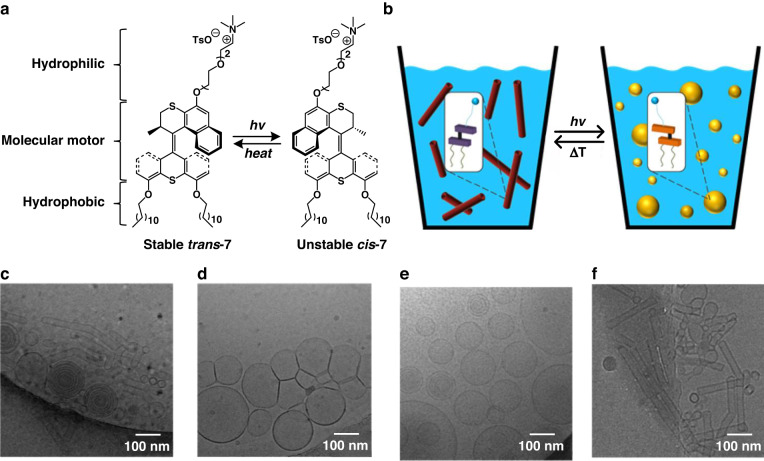
**Reversible self-assembly of amphiphilic molecular motors in aqueous medium**. Schematic illustration of **a** isomerization processes of amphiphilic molecular motor **7** and **b** the corresponding reversible self-assembly transformations between nanotubes and vesicles in aqueous media. Cryo-TEM microscopy images of co-assemblies of amphiphile **7** and DOPC (1:1) in water: **c** before irradiation (stable **7**); **d** after irradiation (unstable **7**); **e** after heating (stable **7**); **f** after freeze−thawing 3 times. Adapted with permission^107^. Copyright © 2015 American Chemical Society

Biological motors often achieve specific biological functions like transport and motion through precise biomolecular assembly in larger systems i.e., membranes and muscles and well-controlled dynamic processes. Inspired by these characteristic phenomena, scientists have employed supramolecular self-assembly to deliver and amplify the movements of molecular motors across length scales to the macroscopic level. In 2016, our group demonstrated the first example of reversible self-assembly of amphiphilic molecular motors in aqueous medium^107^. The amphiphilic molecular motor **7** mixed with 1,2-dioleoyl-*sn*-glycero-3-phosphocholine (DOPC) in water forms well-defined nanotubes, and upon irradiation with UV light, rotary motion of the molecular motor allows the nanotubes to change morphology to vesicles. After a heating and freeze−thaw cycle the system, thermal back isomerization of the molecular motor induces the reformation of the nanotubes (Fig. 4). This study established the foundation for the subsequent development of increasingly complex and highly dynamic artificial nano systems in aqueous media.

**Fig. 5:**
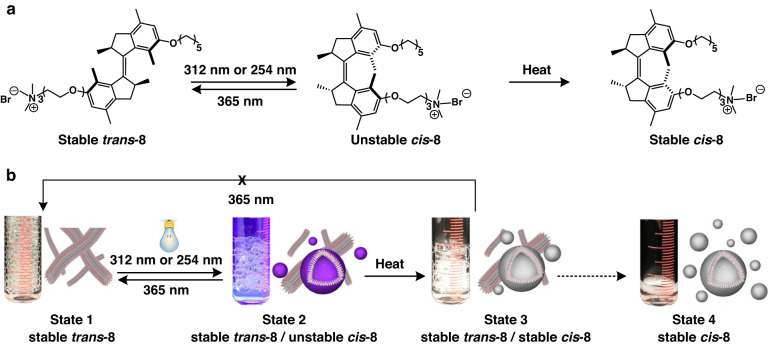
**Control of macroscopic foam properties by dynamic assemblies of molecular motor amphiphiles**. Schematic illustration of **a** the reversible photoisomerization and thermal helix inversion of molecular motor amphiphile **8** and **b** the multi-states of macroscopic foaming processes due to structural transformations in the supramolecular assembly. Reproduced with permission^108^. Creative Commons CC-BY-NC-ND License, 2020, published by American Chemical Society

More recently, our group designed a novel molecular motor-based light-responsive amphiphile **8** that show unique dynamic assemblies featuring multiple states. This allowed the external control of macroscopic foam properties in water^108^. This motor-based responsive supramolecular system provided for unprecedented control over the aggregation behavior, that is, switching from worm-like structures to vesicles and back without helper lipids or extra freeze-thaw cycles solely by rotary motion of the motor, offering new prospects for future soft materials (Fig. 5).

**Fig. 6:**
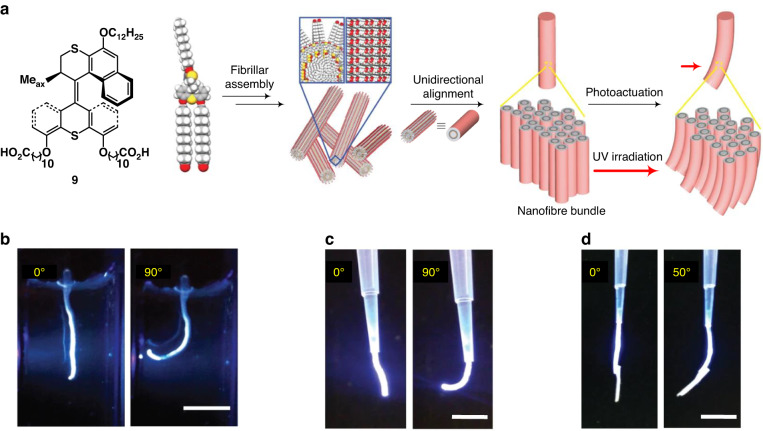
**Macroscopic contractile muscle-like motion of hierarchical self-assembly materials from molecular motors. a** Schematic illustration of the hierarchical supramolecular assemble of amphiphilic molecular motor **9** and its photoactuation behavior. The macroscopic strings were bend toward light source upon UV irradiation in **b** water, **c** air without weight and **d** with 0.4 mg paper as weight. Reproduced with permission^109^. Copyright © 2017 Springer Nature Limited

A major challenge is how to amplify the molecular motion of motors to higher length scales in order to realize macroscopic mechanical motion. In 2018, we took advantage of the hierarchical self-assembly of photoresponsive amphiphilic molecular motors, developed a supramolecular system to perform macroscopic contractile muscle-like motion (Fig. 6)^109^. The amphiphilic molecular motor **9** first assembles into nanofibers with high aspect ratio in water, and it can further align as bundles and form macroscopic strings of centimeter length when muscle-strings were drawn from a CaCl_2_ aqueous solution which, due to the electrostatic interaction between the carboxylate groups and Ca^2+^, induces further precise alignment (Fig. 6a). The strings showed a muscle-like photo-responsive bending motion in either water or air (Fig. 6b, c). The resulted actuation power was enough to lift weight (0.4 mg paper) in air (Fig. 6d). A cooperative mechanism for the photoactuation was proposed and confirmed based on the results of in situ SAXS measurements. Despite the fact that only 5% molecular motor was present in this aqueous soft actuator it underwent robust and repeatable actuation illustrating the power of supramolecular organizational control and amplification of mechanical effects along multiple length scales.

**Fig. 7:**
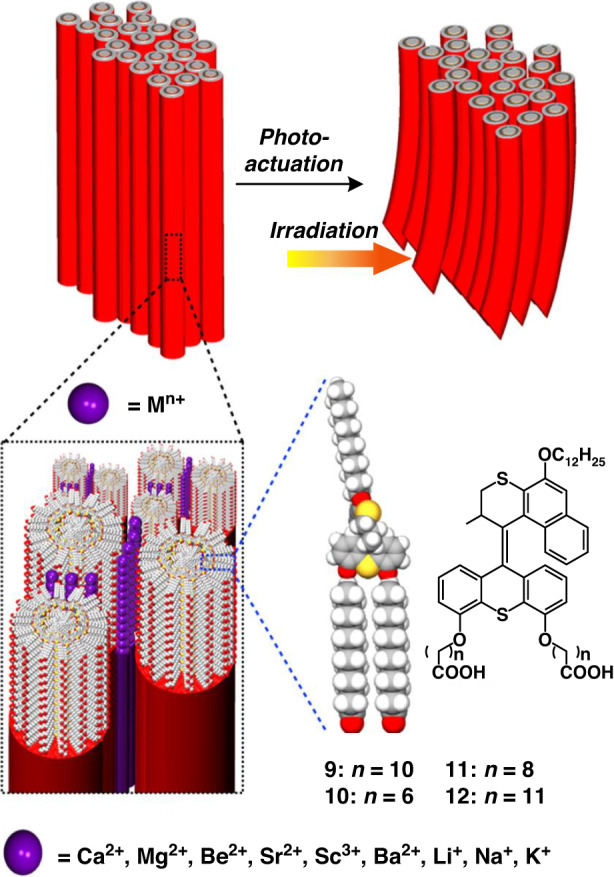
**Molecular structures of molecular motor amphiphiles 9–12 and the hierarchical organization and photoactuation process of their assembled structures in the macroscopic string**. Adapted with permission^110^. Creative Commons CC-BY-NC-ND License, 2018, published by American Chemical Society

Subsequently the effect of the cationic counterions and side chain lengths on nanofiber formation, nanofiber aggregation as well as the packing structure, degree of alignment, and actuation speed of the macroscopic strings were studied (Fig. 7)^110^. It was found that by careful choice of counterions and chain length of molecular motor, it is possible to control the macroscopic motor amphiphiles string structure and achieve tunable actuation speed.

**Fig. 8:**
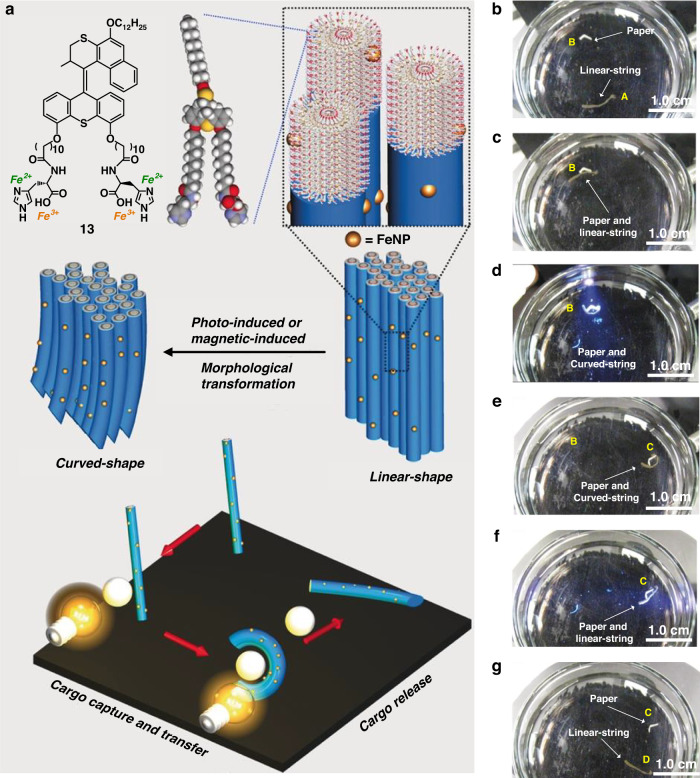
**Light/magnetic field controlled motions of hybrid supramolecular material. a** Schematic illustration of the molecular structure of molecular motor amphiphile **13**, the hierarchical organization and photoactuation and magnetic field induced motions of the assembled structures in the obtained macroscopic string. Snapshots of a dual-controlled cargo transport process in CaCl_2_ solution: **b** the obtained macroscopic string (Position A) and paper (Position B), **c** the string moved to position B, **d** the string changed to a curved-shape upon photoirradiation, **e** the paper was carried to position C by the string which guided by a magnet, **f** the string changed to a linear-shape upon photoirradiation, **g** the paper was unloaded and the string moved to position D. Reproduced with permission^111^. Copyright © 2019 WILEY‐VCH Verlag GmbH & Co. KGaA, Weinheim

In addition, we further looked into the possibility to realize a dual-controlled macromechanical functioning of the unidirectional hierarchical supramolecular structure by incorporating magnetite nanoparticles (Fe_3_O_4_) into the molecular motor based supramolecular nanofibers (Fig. 8)^111^. The string was able to perform a macroscopic cargo transport process under orthogonal controlled stimulation by light and a magnetic field (Fig. 8b–g).

## Molecular motors in polymer networks

**Fig. 9:**
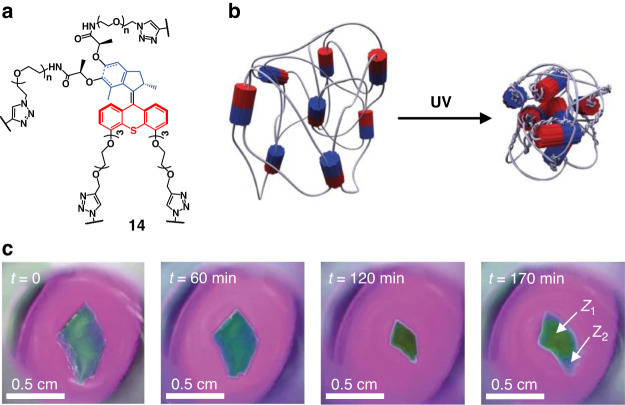
**Macroscopic contraction of molecular motor/polymer gel. a** Chemical structure of molecular motor/polymer conjugates **14**. **b** Schematic representation of contraction of the molecular motor/polymer gel induced by continuous rotation of the motors. **c** Pictures showing time-dependent macroscopic contraction of a piece of polymer gel **14** immersed in toluene and upon UV light irradiation. Adapted with permission^94^. Copyright © 2015, Springer Nature Limited

The use of supramolecular assembly provides an important approach for amplifying molecular motion into macroscopic function. Alternatively, the design of polymeric network with covalently incorporation of motors show promising results as well. In 2015, Giuseppone and co-workers reported a pioneering study on the incorporation of light-driven unidirectional molecular rotors as reticulating units into gel-forming polymer network to induce macroscopic contraction of the material (Fig. 9)^94^. Under UV irradiation, the continuous rotation of the motors actively entangles the polymer chains, ultimately resulting in the contraction of the gel in an isotropic manner. After 2 h of continuous irradiation, the gel shrank to 20% of its original volume. With further extension of the irradiation time, the gel ruptured and recover its initial volume due to the oxidation of the motor double bond (Fig. 9c). In this system, the continuous photoinduced rotation of the motors drives the system to work under far-from-equilibrium conditions, and store energy by converting incident photons into free energy of the entangled polymer chains. Unfortunately, this system was yet irreversible and therefore is limiting its application.

The issue of irreversible behavior was addressed in a subsequent study by introducing an additional diarylethene switch into the crosslinked network, which acts as modulator unit to release the accumulated stress on demand^112^. Upon irradiation with ultraviolet light, the modulator is in its closed form, which can maintain the torsion of the polymer chains produced by the motors rotations and leading to overall contraction of the material. In contrast, when exposed to visible light, the motor stops rotating and the modulator switches back to its open isomer, which generate freely rotating single bonds that can unbraid the polymer chains and thus leads to a re-expansion of the network at thermodynamic equilibrium. It should be noted that the reversibility of the process depends on the elasticity of the braided polymer network and the osmotic pressure. However, as the polymeric gels do not have certain directional alignments inside the material only isotropic contraction are observed. More specific and advanced functions are envisioned with materials with ordered structures that amplify collective motion of molecular motors.

**Fig. 10:**
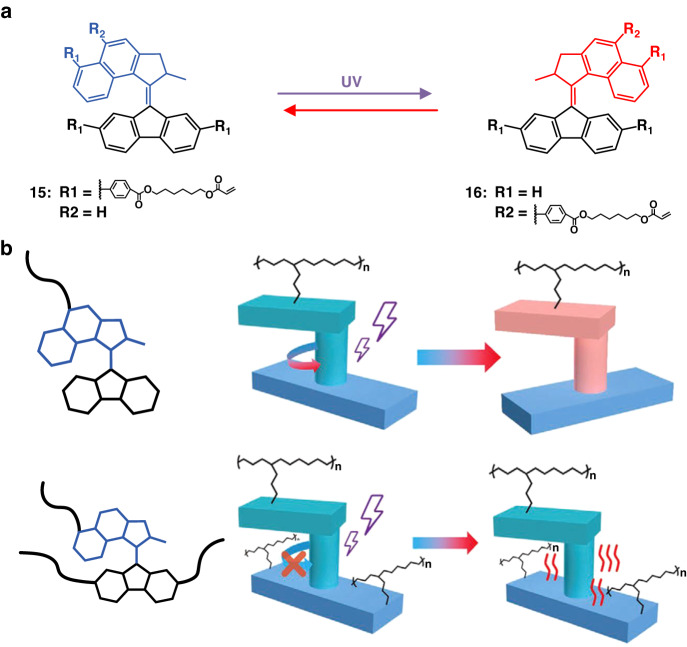
**Light-driven LCNs containing molecular motors. a** Chemical structure of trifunctional molecular motor **15** and monofunctional molecular motor **16**. **b** Schematic illustration of the photo-responsive behaviors of the LCNs cross-linked the molecular motors with different degrees of freedom. Reproduced with permission^118^. Copyright © 2020 WILEY-VCH Verlag GmbH & Co. KGaA, Weinheim

Liquid crystalline networks (LCNs) combine the anisotropy of liquid crystals^113,114^ and the elasticity of polymer network so they are promising polymer materials to amplify the motion of molecular motors^115–117^. In 2020, Yang’s group synthesized trifunctional and monofunctional polymerizable molecular motors with different degrees of freedom, and cross-linked the molecular motors into LCN to prepare novel soft actuators (Fig. 10)^118^. It was found that when the molecular motors act as crosslinking units in the polymer network, the motor was compromised by the polymer chains, and as the rotary motion of molecular motor was constrained, transferring the incident photon energy into heat instead of performing photoisomerization (Fig. 10b).

**Fig. 11:**
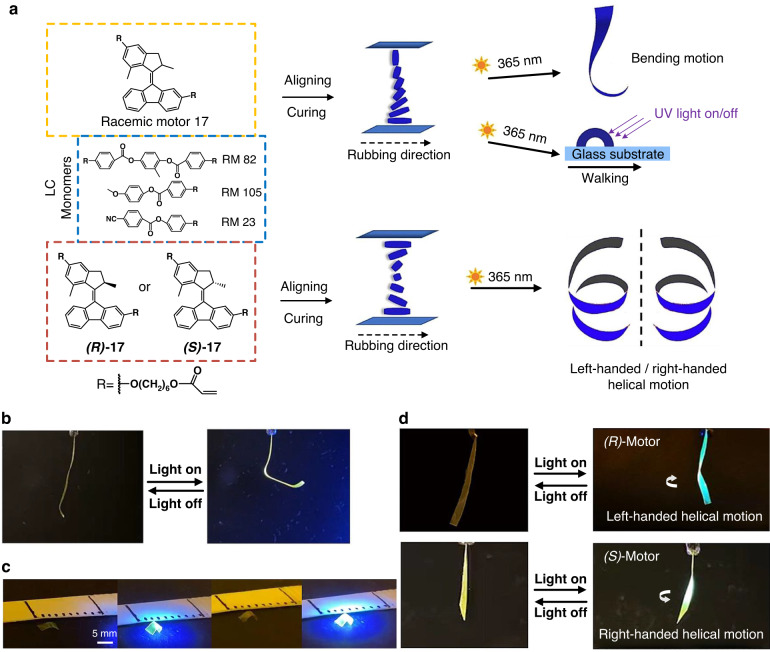
**Photo-responsive helical motion of molecular rotary motor-based liquid-crystal network. a** Chemical structure of the LC monomers and molecular motors for the preparation of a photoresponsive LCN and schematical illustration of photoinduced deformation corresponding LCN film. **b** LCN film crosslinked with racemic motor **17** showed bending motion upon UV irradiation. **c** Photo-induced walking of an LCN film crosslinked with racemic motors on a glass surface. **d** Top: LC ribbons with *(R)*-**17** showed left-handed helical motion upon UV irradiation. Bottom: LC ribbons with *(S)*-**17** showed right-handed helical motion upon UV irradiation. Reproduce with permission^87^. Creative Commons CC-BY 4.0 License, 2021, published by WILEY‐VCH Verlag GmbH & Co. KGaA, Weinheim

In 2021, our group designed a novel molecular motor that can be used as crosslinker, chiral dopant and photo-responsive units in liquid crystal polymer networks (Fig. 11a)^87^. By cross-linking racemic molecular motors into the LCN, the molecular motors can rotate in this system and its rotation and shape change effect the polymer main chains, reduces the order parameter of the mesogenic units which results in the polymer ribbons with splayed alignment show fast bending motion and surface walking upon UV irradiation (Fig. 11a–c). We next used enantiomerically pure motors to study helical motion of LCN polymer materials. To our delight, only 1 wt% the enantiomerically pure motor is required to induce nematic liquid crystals to form cholesteric phases. The polymer films prepared with *R* and *S* chiral motors shown fast right-handed or left-handed helical motion under UV irradiation, respectively (Fig. 11a, d).

**Fig. 12:**
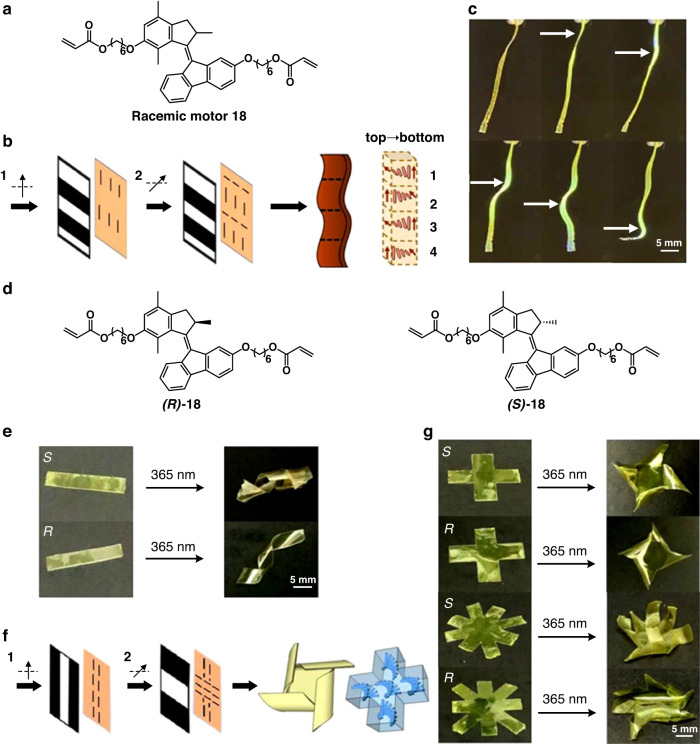
**Phototriggered complex motion by programmable integration of molecular motors in LCNs. a** Chemical structure of racemic motor **18**. **b** Two-step procedure for the preparation of alignment layers by photolithography. The black arrows indicate the polarization direction of the UV light. Before the second exposure step, the sample is rotated at 90°. **c** Photo-induced wavy motion of the polymeric liquid crystal film containing racemic motor **18**. **d** Chemical structure of enantiomerically pure motor *(R)*-**18** and *(S)*-**18**. **e** Photo-induced twisting of polymeric ribbons containing *(R)*-**18** or *(S)*-**18**. **f** Two-step procedure for the preparation of alignment layers by photolithography. **g** Photo-induced helical motion of the polymeric films with different shapes. The UV-light intensity is 230 mw/cm^2^. Reproduce with permission^88^, Creative Commons CC-BY 4.0 License, 2022, published by American Chemical Society

In nature, in addition to simple movements such as bending and spiraling, there are a variety of complex movements. In order to realize the diversity and complex motion of the molecular system in a controllable and adjustable way, in the next step we combined the material with photolithography technology. This allows to programmatically embed the molecular motors into the LCN material as the motors are within a controlled and well-defined orientation in the network (Fig. 12)^88^. The resulting polymeric films with pre-ordering of racemic or homochiral motors can induce not only fast wavy motion (Fig. 12a–c) but also synchronized helical motion with different chirality upon UV irradiation (Fig. 12d, e), enabling complex shape changes and motion on demand, depending on the chirality of the system (Fig. 12f, g). This approach shows how the rotary motion of molecular motors can be programmed in photo-responsive materials and paves the way for the future design of advanced responsive materials with enhanced complex functions.

## Conclusions and outlook

With the elegant design and construction of molecular motors and machines and the demonstration of responsive functions as exemplified with molecular elevators, shuttles, supramolecular actuators, adaptive catalysts, smart pharmaceuticals or even molecular nanocars, a pertinent question in the field of molecular machines is: “could these machines operating at the nanoscale perform work at the macroscopic scale”? It requires that the molecular motion can be amplified effectively in organized systems and soft matter, to produce motion and associated mechanical functions that are not readily achieved otherwise. Organization at interfaces or in confined 3D space or in supramolecular assemblies and cooperativity and amplification along lenght scales from nano- to macro- dimensions is crucial. This review specifically focuses on examples of photo-responsive macroscopic materials based on light-driven molecular motors. Key to successful systems is the collective motion of the unidirectional rotary motors by either confining them on surfaces, in aligned supramolecular environments or in a liquid crystal network where predefined orientation is present. Photo-responsive surfaces show switchable wettability while muscle-like functions are observed in supramolecular materials obtained by hierarchiral assembly of amphiphilic motors in aqueous solution. Light-triggered bending, directional translational movement and orthogonal helical coiling of polymeric liquid crystal strings are demonstrations of precise mechanical movements at the macro-scale by embedding rotary motors in liquid crystal networks. The mechanical systems discussed in this review illustrate, and enhance our understanding, how complex movement can be programmed in light-responsive materials. As shown here, besides the traditional photo-switches including azobenzene and diarylethylene, light-powered molecular motors offer ample opportunities to develop man-made adaptive and dynamic materials, which can serve to provide important guidelines for the future design of more advanced and multifunctional responsive materials that can perform elaborate and complex tasks.
